# Site-selective modifications by lipid A phosphoethanolamine transferases linked to colistin resistance and bacterial fitness

**DOI:** 10.1128/msphere.00731-24

**Published:** 2024-11-29

**Authors:** Anna Schumann, Ahmed Gaballa, Hyojik Yang, Di Yu, Robert K. Ernst, Martin Wiedmann

**Affiliations:** 1 Department of Food Science, Cornell University 5922 , Ithaca, New York, USA; 2 Graduate Field of Biomedical and Biological Sciences, Cornell University 5922 , Ithaca, New York, USA; 3 Department of Microbial Pathogenesis, School of Dentistry, University of Maryland Baltimore 12265 , Baltimore, Maryland, USA; University of Nebraska Medical Center College of Medicine, Omaha, Nebraska, USA

**Keywords:** antimicrobial resistance, colistin, polymyxins, lipopolysaccharide, phosphoethanolamine, mcr

## Abstract

Genes encoding lipid A modifying phosphoethanolamine transferases (PETs) are genetically diverse and can confer resistance to colistin and antimicrobial peptides. To better understand the functional diversity of PETs, we characterized three canonical mobile colistin resistance (*mcr*) alleles (*mcr-1*, *-3*, *-9*), one intrinsic *pet* (*eptA*), and two *mcr*-like genes (*petB*, *petC*) in *Escherichia coli*. Using an isogenic expression system, we show that *mcr-1* and *mcr-3* confer similar phenotypes of decreased colistin susceptibility with low fitness costs. *mcr-9*, which is phylogenetically closely related to *mcr-3*, and *eptA* only provide fitness advantages in the presence of sub-inhibitory concentrations of colistin and significantly reduce fitness in media without colistin. PET-B and PET-C were phenotypically distinct from bonafide PETs; neither impacted colistin susceptibility nor caused considerable fitness cost. Strikingly, we found for the first time that different PETs selectively modify different phosphates of lipid A; MCR-1, MCR-3, and PET-C selectively modify the 4′-phosphate, whereas MCR-9 and EptA modify the 1-phosphate. However, 4′-phosphate modifications facilitated by MCR-1 and -3 are associated with lowered colistin susceptibility and low toxicity. Our results suggest that PETs have a wide phenotypic diversity and that increased colistin resistance is associated with specific lipid A modification patterns that have been largely unexplored thus far.

**IMPORTANCE:**

Rising levels of resistance to increasing numbers of antimicrobials have led to the revival of last resort antibiotic colistin. Unfortunately, resistance to colistin is also spreading in the form of *mcr* genes, making it essential to (i) improve the identification of resistant bacteria to allow clinicians to prescribe effective drug regimens and (ii) develop new combination therapies effective at targeting resistant bacteria. Our results demonstrate that PETs, including MCR variants, are site-selective in *Escherichia coli* and that site-selectivity correlates with the level of susceptibility and fitness costs conferred by certain PETs. Site selectivity associated with a given PET may not only help predict colistin resistance phenotypes but may also provide an avenue to (i) improve drug regimens and (ii) develop new combination therapies to better combat colistin-resistant bacteria.

## INTRODUCTION

Gram-negative bacteria are becoming more resistant to available antimicrobials. Hence, last-resort antimicrobials, including colistin (polymyxin E), are increasingly used as therapeutics (1, 2). Colistin resistance (col^R^), which the Clinical Laboratory and Standards Institute (CLSI) defines as the ability of bacteria to grow in colistin concentrations above a breakpoint (i.e., 2 µg/mL) (3), has been increasing, which is driven through the spread of mobile colistin resistance (*mcr*) genes (4). MCR variants encoded by these genes are part of a group of enzymes specific to Gram-negative bacteria, classified as lipid A phosphoethanolamine transferases (PETs), which catalyze the transfer of phosphoethanolamine (pEtN) to one of the terminal phosphate moieties of lipid A (5, 6). This modification reduces the overall negative charge of the outer membrane, decreasing colistin’s ability to interact with the negatively charged phosphates of lipid A (7). To date, 10 different MCR families encompassing 115 different alleles have been discovered; members of these MCR families share varying degrees of amino acid sequence similarity with themselves and the intrinsic PET, EptA (4, 5). Although MCR variants are well known for providing col^R^, recent studies have reported that MCR expression can lead to fitness costs (8–10), lead to potential cross-resistance to other antimicrobial peptides (AMPs) (11–13), or impact bacterial-host interactions (11, 14). Additionally, it seems that the ability of individual MCR variants to confer col^R^ differs, which is currently being evaluated further (15). For example, MCR-9 was shown to decrease the susceptibility to colistin when it was first discovered (16), and the ability to confer col^R^ was confirmed when *Enterobacter cloacae* isolates were tested in non-standard minimum inhibitory concentration (MIC) assay media (17). However, several studies showed that isolates that naturally carry MCR-9 were susceptible in standard MIC assays (18, 19). This demonstrates that the functional diversity of MCR and PET variants is scarcely understood.

To quantitatively probe the phenotypic diversity of MCR variants, as well as PETs that have been linked to the stress response to AMPs (20), we optimized a standardized and monitorable expression system to physiologically and biochemically characterize a range of canonical (i.e., EptA, MCR-1, -3, -9) and novel PETs (i.e., PET-B, -C). PET-B and -C were identified as part of two putative, novel MCR families based on amino acid sequence similarities with previously identified MCR and PET alleles (5); the genes encoding for these novel PETs are both located on *Pseudomonas aeruginosa* chromosomes. Overall, we found that PET families represent diverse phenotypes and that PETs show stereoselective pEtN modifications of lipid A in *E. coli*, which may be associated with col^R^ phenotypes and may provide avenues for the development of new antibiotic adjuvants and drugs.

## RESULTS

### Cell viability defects and colistin MICs of canonical PETs differ

**Fig 1:**
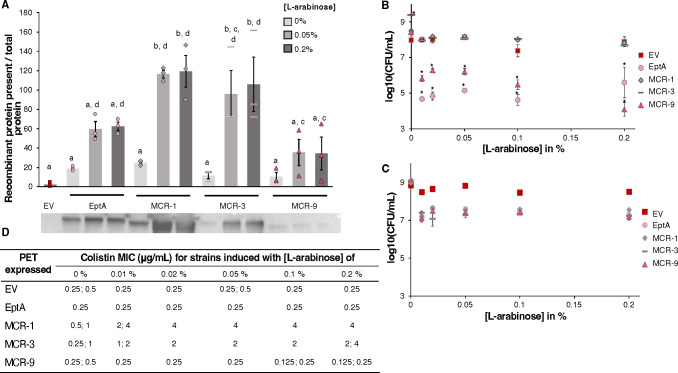
Characterization of *E. coli* Top10 expressing canonical PET variants induced with different L-arabinose concentrations. For all experiments, strains were grown in MHII media for 2 h at 37°C, 200 rpm prior to induction. (**A**) Western blotting of cells was performed on samples that were collected after 12 h of induction with 0.05% or 0.2% of L-arabinose; anti-FLAG antibodies were used to detect heterologously expressed FLAG-tagged PET proteins, data represent means, *n* = 3 ± SEM, samples with the same letter are not significantly different, using a one-way ANOVA and post-hoc Tukey test. Higher ratios on the y-axis correspond to stronger signals and higher recombinant protein levels. (**B, C**) Cell viability was assessed after 4 (**B**) and 12 h (**C**) of incubation with different L-arabinose concentrations, data represent means, *n* = 3 ± SEM, * indicates significant differences from the EV control at the same concentration (α = 0.05) using emmeans after a two-way ANOVA. (**D**) Colistin MICs of strains added to plates after 1 h of induction with different L-arabinose concentrations; MIC assays were performed in biological triplicates; two concentrations were reported if experiments yielded two different MICs for a given strain.

To better characterize the phenotypic diversity of PETs, we created a standardized expression system that includes the colistin-susceptible strain *E. coli* Top10 (21) and the vector pBAD24 with an L-arabinose-inducible promoter. Four canonical *pet* genes (i.e., *mcr-1*, *mcr-3*, *mcr-9*, and *eptA*) were cloned into this expression system, and protein expression was confirmed for uninduced and induced strains using western blots (Fig. 1A; Fig. S1). Although MCR-1 and MCR-3 protein levels were significantly higher when induced with either 0.05% or 0.2% of L-arabinose in comparison to uninduced controls (*P* < 0.01), there was no significant difference in EptA and MCR-9 protein levels between induced and uninduced strains (Fig. 1A). Interestingly, MCR-9 protein levels were significantly lower compared with (i) MCR-1 levels for samples induced with 0.05 or 0.2% L-arabinose (*P* < 0.01) and (ii) MCR-3 levels for samples induced with 0.2% L-arabinose (*P* < 0.05) (Fig. 1A).

To identify an optimal L-arabinose concentration to induce PET expression, we measured cell viability and colistin MICs for strains expressing canonical PETs, in comparison to an empty vector control (“EV”) at different inducer concentrations (Fig. 1; Fig. S2). After induction for 4 h, the cell viability of strains expressing MCR-1 or -3 was not significantly different from the EV across all inducer concentrations (Fig. 1B; Fig. S2). In contrast, strains expressing EptA or MCR-9 showed significant reductions in cell viability (relative to the EV) across all inducer concentrations (*P* < 0.05); for example, MCR-9 and EptA expressing strains showed mean cell viability reductions of 1.9 and 3.0 log, respectively, when induced with 0.05% L-arabinose (Fig. 1B).

Inducer concentrations also impacted colistin MICs. For all strains, 0.02% or 0.05% L-arabinose yielded maximum MIC levels, which were (i) 0.25 µg/mL for the EV, EptA, and MCR-9, (ii) 2 µg/mL for MCR-3, and (iii) 4 µg/mL for MCR-1 expressing strains (Fig. 1D). Hence, we selected 0.05% L-arabinose as our standardized expression condition for further experiments because this concentration resulted in (i) maximum fold changes in colistin susceptibility in comparison to the EV, (ii) minimum cell viability defects, and (iii) reliable protein expression across all our PETs (Fig. 1).

### Novel *mcr*-like PETs are phenotypically distinct from canonical PETs

**Fig 2:**
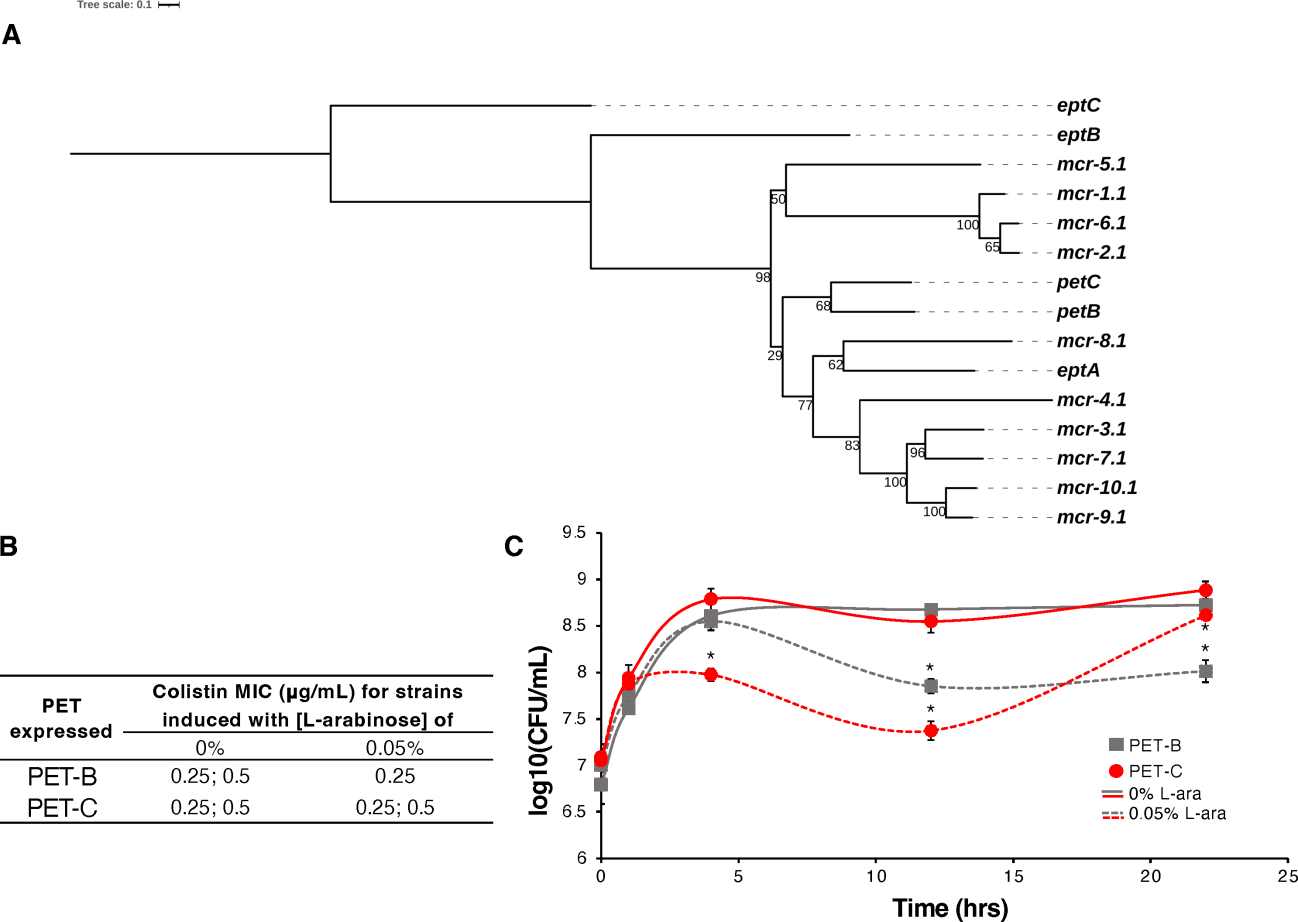
Characterization of novel, *mcr-*like PETs in *E. coli* Top10. (**A**) Phylogenetic tree shows the relatedness of novel, *mcr*-like *pet* genes to chromosomally encoded *pet* genes (i.e., *eptA*, *eptB*, and *eptC*) and *mcr* variants. (**B**) Colistin MICs were obtained after growth in MHII broth for 2 h, followed by induction with 0 or 0.05% of L-arabinose for 1 h. MIC assays were performed in biological triplicates; two concentrations were reported if experiments yielded two different MICs for a given strain. (**C**) Cell viability was determined at several time points after induction with 0% or 0.05% of L-arabinose, data represent means, *n* = 3 ± SEM, * indicates significant differences between the uninduced, isogenic control (α = 0.05) based on emmeans after a two-way ANOVA.

We previously used bioinformatics approaches to predict novel *mcr*-like genes that may constitute new MCR families (5). To further characterize the diversity of PETs, we characterized two of those predicted putative, novel, chromosomally encoded *mcr*-like PETs (i.e., PET-B, -C) (Fig. 2A; see Materials and Methods for a detailed description on the selection of variants). Western blot analysis confirmed that both proteins were expressed (Fig. S3). Colistin MICs of *E. coli* expressing PET-B and -C were similar for uninduced and induced (0.05% L-arabinose) strains (MICs of 0.25–0.5 µg/mL; Fig. 2B); these MIC values were similar to the EV control (Fig. 1D). We however found evidence that expression of PET-B and -C conferred some cytotoxicity; compared with uninduced controls, strains expressing PET-B showed mean log reductions in cell viability of 0.8 and 0.7 log after 12 and 22 h but not after 4 h, whereas strains expressing PET-C showed 0.8 and 1.2 log reductions after 4 and 12 h of induction (*P* < 0.05) (Fig. 2C).

### PET-expressing strains differ in their response to various cell envelope stressors

As MCR-1 has been shown to provide cross-resistance to AMPs (11, 13, 22), we characterized the ability of different PETs to lower susceptibility to different AMPs and cell envelope stressors. Strains expressing EptA or MCR-9 showed considerably lower MICs for EDTA (a metal chelator) (4 and 2 mg/mL, respectively) compared with strains expressing any other PET or EV (MIC of 16 mg/mL) (Table 1). We performed zone of inhibition (ZOI) assays for the other cell envelope stressors due to the limited availability of these compounds in pure form. In ZOI assays, colistin and cecropin A, a positively charged AMP (23), were the only compounds that yielded visible ZOIs (Table 1). Although the EV and the PET-B expressing strain had the largest ZOIs for colistin (15 and 14.75 mm, respectively) and cecropin A (12 mm), ZOIs did not substantially differ between the other PET-expressing strains (e.g., cecropin A ZOIs ranged from 11 to 11.5 mm for PET expressing strains other than PET-B).

**TABLE 1:** Zone of inhibitions and MIC values of *E. coli* Top10 strains expressing PET variants, induced with 0.05% L-arabinose to antimicrobial peptides or cell envelope stressors

PET expressed	Zone of inhibition (mm)^*a*^ for:		MIC (mg/mL)^*b*^ for EDTA
	Colistin	Cecropin A	
EV^*c*^	15	12	16
EptA	14	11	4
MCR-1	13	11.5	16
MCR-3	13.5	11.25	16
MCR-9	14	11	2
PET-B	14.75	12	16
PET-C	14	11.25	16

^*a*^ Zone of inhibitions represents means of biological duplicates. Substances were added to disks in 10 μL volumes for a final amount of 10 μg or 1% for SDS. Negative controls (i.e., H_2_O and acidic H_2_O [pH = 2]) showed no zone of inhibition. LL-37, SDS, and nisin showed no zone of inhibition in any strains.

^*b*^ MIC was determined from biological triplicates and represents the most frequent MIC.

^*c*^ “EV” = empty vector control.

### PETs confer context-dependent fitness advantages and disadvantages

We further characterized our PET variants by performing (i) 1-h killing assays and (ii) competition assays. Compared with static MIC values, both assays allow for better assessments of the dynamic nature of bacterial interactions with antibiotics (24).

**Fig 3:**
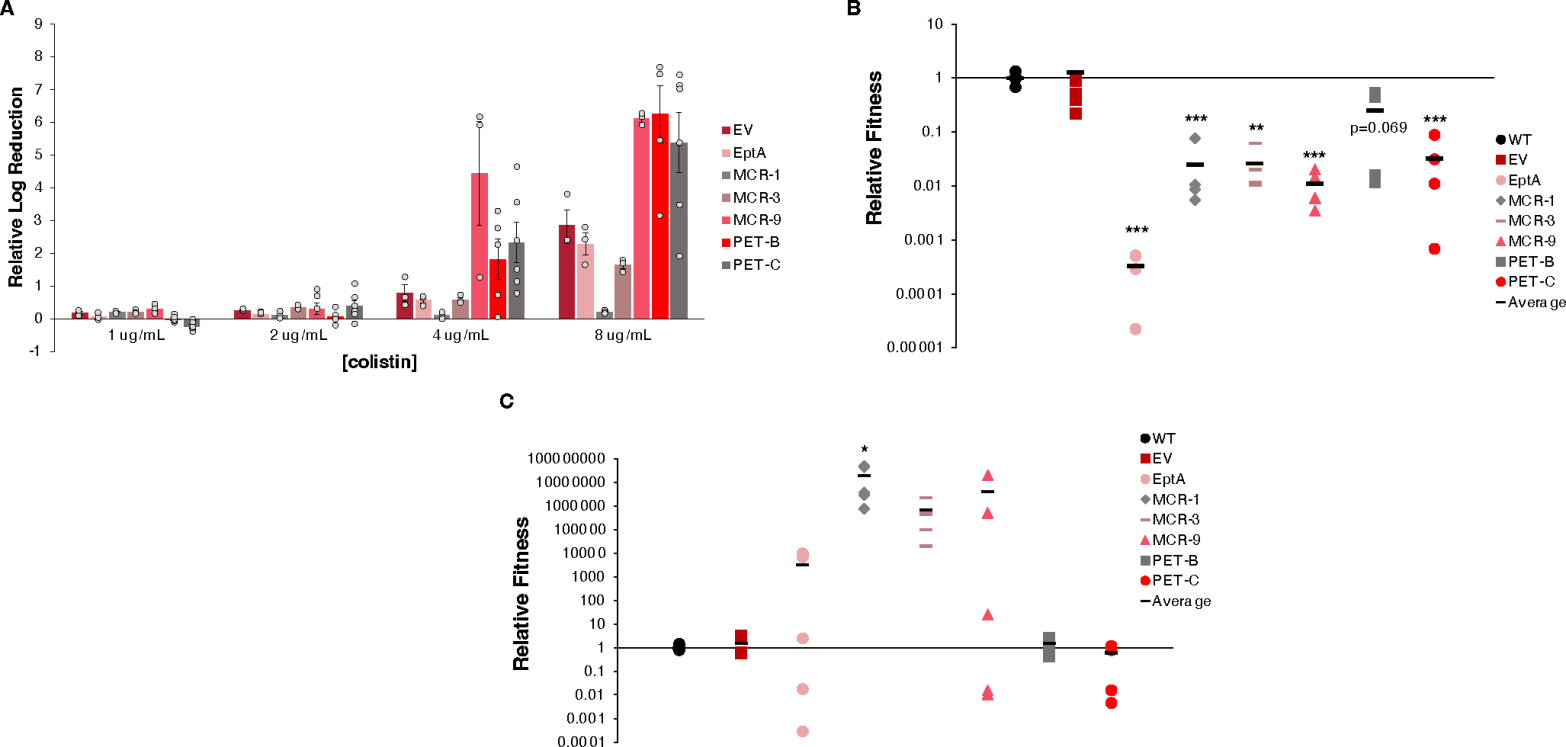
Colistin resistance and fitness of PET expressing *E. coli* Top10 strains. For all data shown, strains were grown for 2 h at 37°C and induced with 0.05% L-arabinose for 1 h prior to experiments. (**A**) Relative log reduction of *pet* expressing strains after exposure to different colistin concentrations in 1 h killing assays in comparison to strains grown in the absence of colistin, data represent means, *n* = 3 ± SEM, except *n* = 5 for PET-B and *n* = 6 for PET-C. (**B**) Fitness of *pet* expressing strains (*lacZ^-^*) after 12 h competition with *E. coli* pUC19 (*lacZ^+^*) strains in MHII without colistin. Relative fitness was calculated by adjusting fitness values by the average fitness of the WT strain for each experiment, black bars represent means, *n* = 4, and significance values are based on comparison to WT strains using a one-way ANOVA and post hoc Dunnett’s test. (**C**) Fitness of *pet* expressing strains (*lacZ^-^*) after 12 h competition with *E. coli* pUC19 (*lacZ^+^*) strains in MHII in the presence of colistin (concentrations at ¼ of each strain’s colistin MIC). Fitness values are relative to the WT strain, black bars represent means, *n* = 5, and significance values are based on comparisons to the WT strains using a Kruskal-Wallis test and post-hoc many-to-one Dunn’s test. *, *P* < 0.05; **, *P* < 0.01, ***, *P* < 0.001.

For killing assays, exposure to colistin was performed for 1 h; this time frame has been previously used to identify phenotypic differences between MCR variants (16). To measure die-off dynamics of our PET expressing strains at concentrations around the colistin breakpoint (i.e., 2 µg/mL), we performed killing assays at colistin concentrations ranging from ¼× to 2× the colistin breakpoint using 2-fold dilution steps. Overall, higher colistin concentrations corresponded to higher log reductions for the EV and all PET*-*expressing strains except MCR-1. For all strains (except MCR-1), the relative log reduction was <1 log after exposure to 1 and 2 µg/mL of colistin but increased to 1.6–6.2 log after exposure to 8 µg/mL of colistin (Fig. 3A). Strains expressing MCR-1 showed consistent tolerance to colistin (<0.2 log reduction at all tested colistin concentrations). Differences between PETs were most apparent at 8 µg/mL of colistin: both MCR-1 (0.2 log reduction) and MCR-3 (1.6 log reduction) conferred increased tolerance compared to the EV (2.9 log reduction). Although the EptA strain showed a 2.3 log reduction (similar to EV), MCR-9, PET-B, and PET-C showed substantially increased killing (6.1, 6.2, and 5.4 log, respectively) relative to the EV (Fig. 3A).

Competition assays were used to assess fitness by determining the relative changes in cell numbers of two strains grown in co-cultures (25). Competition assays were performed in the presence and absence of colistin to understand the context-dependent fitness costs of PET expression. Here, fitness is defined as the ratio of surviving PET-expressing cells to surviving competitor cells (*E. coli* Top10 pUC19), normalized by their initial levels. In the absence of colistin, the expression of different PETs conferred varying degrees of fitness costs; strains expressing (i) EptA were recovered at >1,000 fold lower levels (*P* < 0.001), (ii) MCR-1, PET-C, MCR-9 (*P* < 0.001), and MCR-3 (*P* < 0.01) were recovered at 10-fold to 100-fold lower levels, and (iii) PET-B was recovered at <10-fold lower levels (*P* = 0.069) than the competitor strain (Fig. 3B).

*E. coli* expressing canonical PETs were considerably more fit than the competitor strain (Fig. 3C) in the presence of colistin at one-fourth of the PET-expressing strain’s MIC (Fig. 1D and 2B). Strains expressing EptA were recovered at 100-fold, MCR-1 at 10^7^-fold (*P* < 0.05), MCR-3, and MCR-9 at 10^6^-fold higher levels than the competitor strain. In contrast, strains expressing PET-B or -C were recovered to the same extent as the competitor strain (Fig. 3C). Interestingly, the competitive indices of EptA (*P* < 0.01) and MCR-9 (*P* < 0.05) expressing strains varied significantly more when compared with all other strains (except for the comparison between variances for EptA and PET-C expressing strains).

### PET enzymes are stereoselective in their lipid A modification

**Fig 4:**
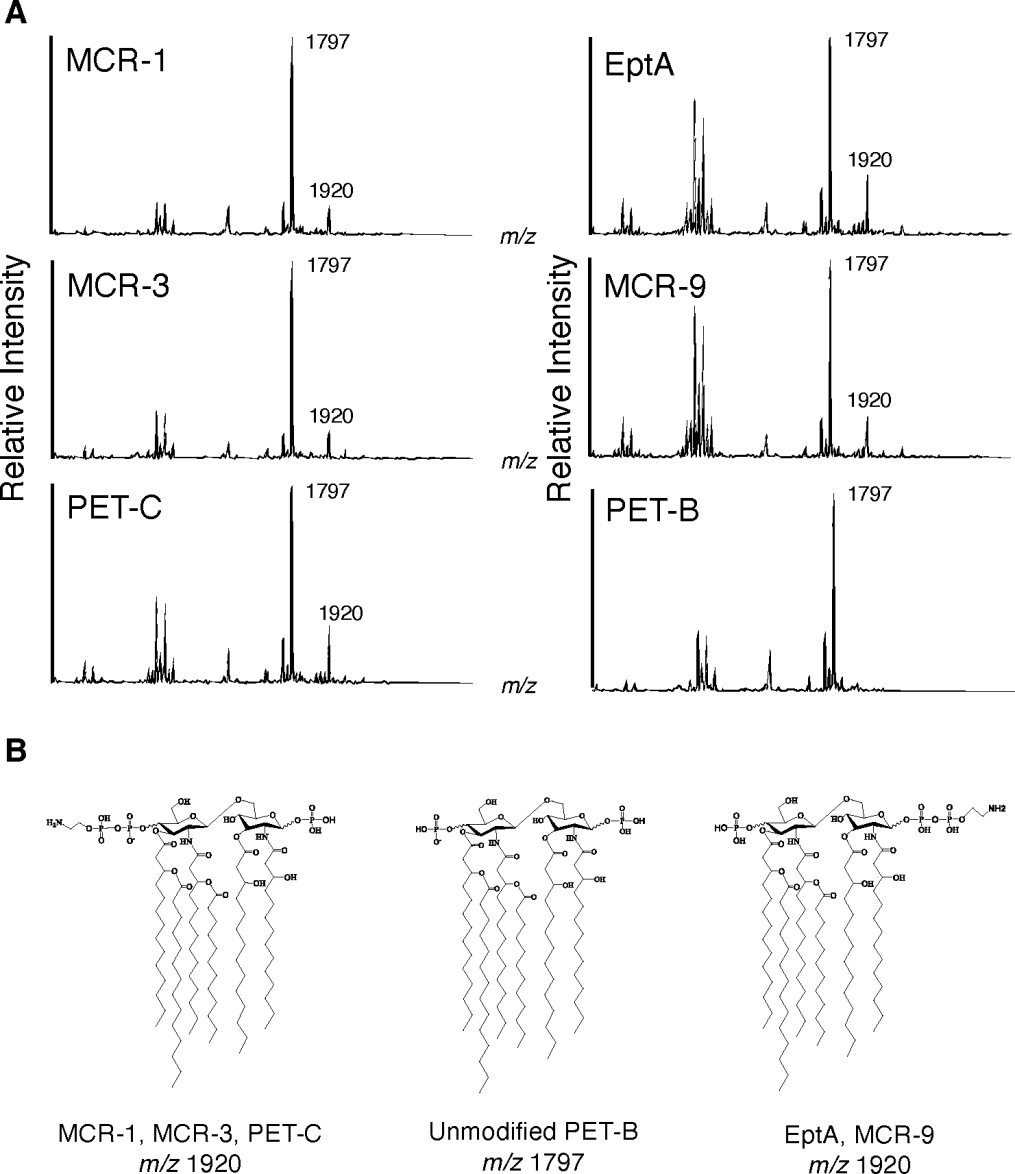
Site-selective modification of lipid A by PETs. FLAT mass spectra and chemical structure of lipid A. (**A**) *E. coli* strains expressing five different PETs (MCR-1, MCR-3, PET-C, EptA, and MCR-9) show lipid A signals at *m/z* 1,797 and 1,920, whereas the PET-B-expressing *E. coli* strain only shows a lipid A signal at *m/z* 1,797. (**B**) Chemical structure of each lipid A molecule; MCR-1, MCR-3, and PET-C have 4’-phosphate group modification with pEtN, EptA and MCR-9 have 1-phosphate group modification. PET-B does not show pEtN modification. All structural analyses were conducted by tandem MS (FLAT^n^), as shown in Fig. 5.

We confirmed the functionality of our heterologous proteins by assessing pEtN-modification of lipid A 12 h after induction with 0.05% L-arabinose. The detected ions at *m/z* 1,797 and 1,920 indicated hexa-acylated lipid A and pEtN-modified hexa-acylated lipid A (∆123 *m/z*), respectively (Fig. 4A). PEtN modifications were detected for strains expressing EptA, MCR-1, MCR-3, MCR-9, and PET-C, but not for the EV or PET-B expressing strains (Fig. 4A; Table S1).

**Fig 5:**
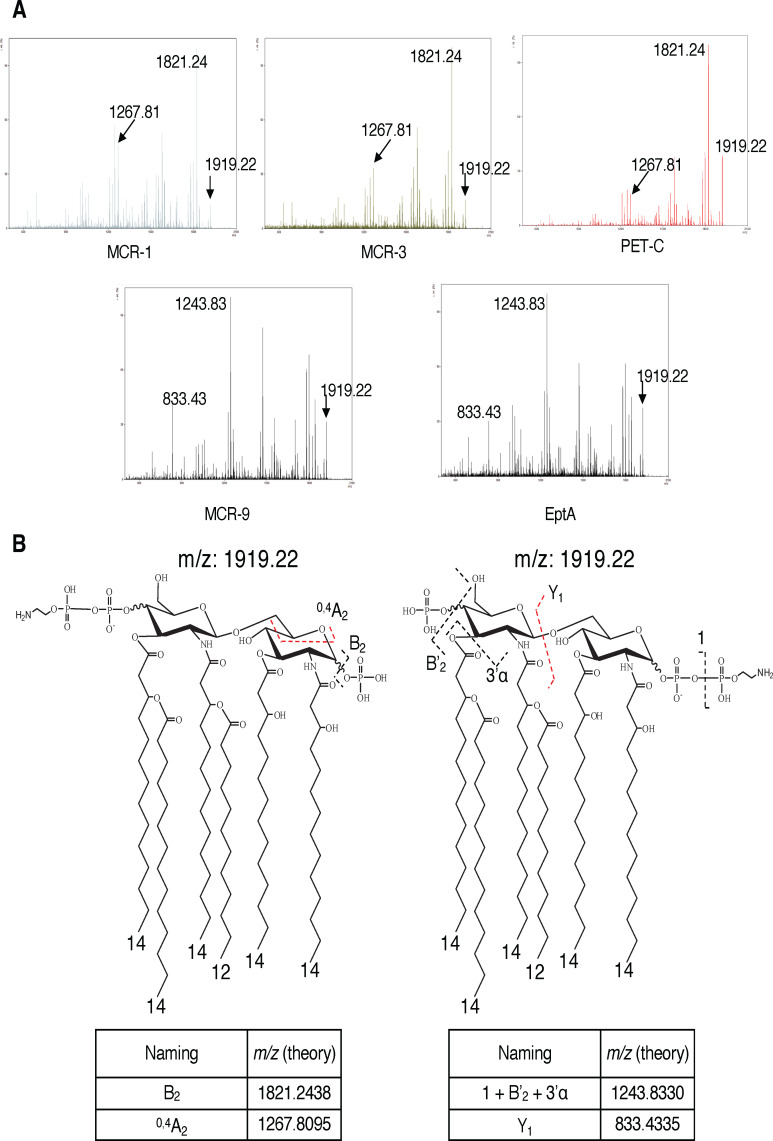
pEtN positions determination by FLATn. FLATn conducted the structural analysis of pEtN-modified lipid A at *m/z* 1,919.22. (**A**) *E. coli* strains expressing five different PETs (MCR-1, MCR-3, PET-C, MCR-9, and EptA) were investigated to determine which site is pEtN-modified. Three strains (MCR-1, MCR-3, and PET-C) show similar fragmentation patterns and have *m/z* 1,821.24 as a base peak and a diagnostic ion at *m/z* 1,267.81, which indicates the modification site by pEtN is the 4′-phosphate. Two other strains (MCR-9 and EptA) show different fragments compared to MCR-1, MCR-3, and PET-C. The base peak and diagnostic fragment in FLATn mass spectra of MCR-9 and EptA are *m/z* 1,243.83 and 833.43, indicating that the modification site by pEtN is the 1-phosphate. (**B**) Chemical structures of the modified lipid A on the phosphate group with pEtN are shown; the fragments and their theoretical *m/z* values are listed below the chemical structures.

To determine the stereospecificity of individual PETs, tandem mass spectra were generated with MS/MS patterns showing two structural isomers from the precursor ion at *m/z* 1,919.22, allowing for the determination of the pEtN location (26). MCR-1, MCR-3, and PET-C showed a fragmentation pattern with a base peak at *m/z* 1,821.24 and a diagnostic ion of 4′-phosphate modification with pEtN at *m/z* 1,267.81 (Fig. 5A). These ions originate from the loss of 1-phosphate and cross-ring cleavage (^0,4^A_2_), respectively (Fig. 5B). In contrast, EptA and MCR-9 showed a fragmentation pattern of the precursor ion at *m/z* 1,919.21 with a base peak at *m/z* 1,243.83 and a diagnostic ion of 1-phosphate modification with pEtN at *m/z* 833.43 (Fig. 5A). These ions originate from multiple neutral losses of an acyl chain, 4-phosphate, and pEtN (1 + B′2 + 3′α) and glycosidic bond dissociation (Y1), respectively (Fig. 5B). The ratio of intensities of the two diagnostic ions at *m/z* 833.43 (1-phosphate modification) and 1,267.81 (4′-phosphate modification) indicated that MCR-1, MCR-3, and PET-C selectively attach the pEtN residue to the 4′-phosphate with 97%, 98%, and 97% efficacy, respectively (Fig. S4; Fig. 4). In contrast, EptA and MCR-9 preferentially attach the pEtN residue to the 1-phosphate with 75% efficacy each (Fig. S4; Fig. 4B).

### PET-B’s extended loop region differs structurally from other PETs

**Fig 6:**
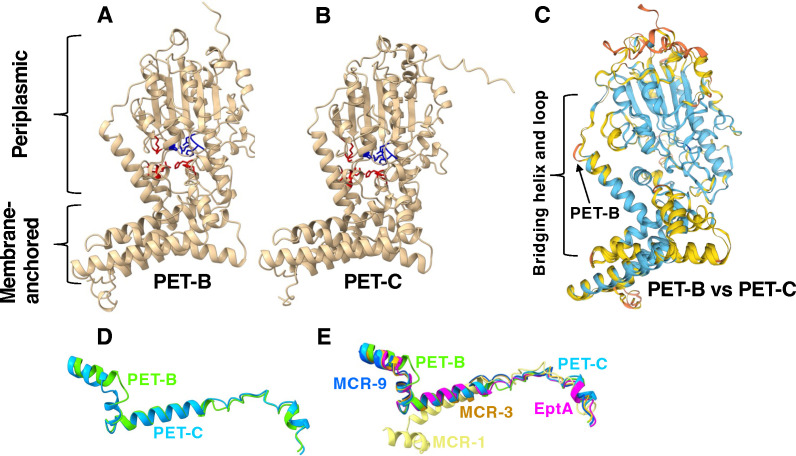
PET-B and PET-C share conserved domain architecture with localized structural variations. (**A**) PET-B and (**B**) PET-C structural models were predicted using AlphaFold2 based on the *N. meningitidis* phosphoethanolamine transferase EptA structure as a template (NCBI structure accession number 5FGN). Structures were viewed and edited using UCSF ChimeraX. Structural models show the conservation of distinct transmembrane-anchored and soluble periplasmic domain architecture and AA residues involved in forming the catalytic (blue residues) and pEtN binding site (red residues). (**C**) A superimposed image of the PET-B and PET-C structures was constructed using Swiss-Model structural comparison tools, showing regions with low similarity confidence (yellow) and very low similarity confidence (orange). (**D**) Localized structural variability of the bridging helix and extended loop regions of PET-B (light green) and PET-C (light blue). PET-B and PET-C structural comparisons were performed using the Swiss-Model server, and the superimposed structures were downloaded, viewed, and edited using UCSF ChimeraX. (**E**) Localized structural variability of the bridging helix and extended loop regions of PET-B (light green), PET-C (light blue), EptA (magenta), MCR-1 (yellow), MCR-3 (orange), and MCR-9 (blue). Structural comparisons were performed using the Swiss-Model server, and the superimposed structures were downloaded, viewed, and edited using UCSF ChimeraX.

Consistent with previous reports (16), our structural models showed that PETs share a highly conserved overall domain structure with discretely folded N-terminal membrane-anchored and C-terminal soluble periplasmic domains (Fig. 6A and B). The structural similarities dendrogram and the correspondence analysis of the structural similarity matrix showed that PETs formed two distinct groups, including (i) MCR-1, -2, and -6, and (ii) EptA, PET-B, PET-C, and the remaining MCR variants (including MCR-3 and -9) (Fig. S5). The modeled PET-B and -C structures showed similar domain architectures and conserved residues involved in the catalytic and pEtN binding sites (Fig. 6A and B). However, superimposed structural models indicated that structures of a periplasmic loop separating two membrane-anchored alpha-helices, as well as the bridging alpha-helix, and extended loop connecting the membrane-anchored and periplasmic domains, differ substantially between PET-B and -C (Fig. 6C and D). Although the bridging helix and extended loop are conserved among PET-C, EptA, MCR-3, and MCR-9 (Fig. 6E), they are markedly different between MCR-1 and other PET-predicted structures (Fig. 6E; Fig. S5).

## DISCUSSION

Here, we used a standardized expression system to functionally and biochemically characterize six distinct *pet* genes, including (i) two widely distributed *mcr* variants with a well-supported ability to confer col^R^ (*mcr-1* and *mcr-3*); (ii) *mcr-9*, a *mcr* gene with a disputed ability to confer col^R^; (iii) *eptA*, an intrinsic *pet* involved in the bacterial stress response to AMPs; and (iv) two chromosomally encoded uncharacterized *pet* genes (*petB* and *petC*, referred to as “*mcr*-like PETs”). Our data indicate that known MCR variants (i.e., MCR-1, -3, and -9) differ in their ability to confer col^R^. Although the expression of the three MCR variants incurs a range of fitness costs in the absence of colistin, the expression of these MCR variants appears to provide variable fitness advantages in the presence of subinhibitory levels of colistin. We also found that the two *mcr*-like PETs characterized here included one gene (*petC*) that modifies lipid A in *E. coli* and one gene (*petB*) that had no detectable phenotype in *E. coli*; these results may be explained by the fact that these genes have primarily been identified in *P. aeruginosa* (5), a species in which *mcr* genes are not commonly identified in (27) and which may have a different lipid A structure and use different lipid A modification enzymes (28, 29). Most strikingly, we found that MCR-1 and -3, which considerably lowered susceptibility to colistin, stereoselectively modify lipid A on the 4′-phosphate, whereas EptA and MCR-9 modify the 1-phosphate.

Although we established an optimized expression system with a fine-tuned induction concentration, previous studies using similar expression strains typically used a higher L-arabinose concentration (i.e., 0.2%), which caused greater cellular defects that might impact colistin susceptibility; however, these studies usually did not assess how those higher inducer concentrations impacted susceptibility to colistin (8, 9, 30). Using our *E. coli* expression system, we found that MCR-1 and -3 lowered susceptibility to colistin 8- and 16-fold (i.e., MICs of 4 and 2 µg/mL, respectively). Wild-type strains carrying these genes typically showed similar MICs, e.g., MICs of 4–8 μg/mL for *E. coli* carrying either *mcr-1* or *-3* (31, 32). Killing assay data suggested phenotypic differences between MCR-1 and -3, with MCR-1 providing protection at higher colistin concentrations, thus also contributing to colistin tolerance. We also showed that MCR-1 and -3 expression conferred the lowest fitness costs, which suggests that these two PETs have evolved to provide effective protection against colistin with minimal impact on cell growth. This is consistent with previous observations that fitness costs associated with *mcr-1* expression are rarely detected in transconjugants carrying natural *mcr*-expressing plasmids (33, 34) and the fact that *mcr-1* and *-3* represent the most diverse *mcr* families with 37 and 42 variants, respectively (NCBI Pathogen Detection, Reference Catalog, 10/02/24). However, it has also been shown that bacterial hosts acquire compensatory mutations in their genomes, which decrease the fitness burden of *mcr* carriage (10, 35). Our study does not examine how changes within the host’s genome may impact *mcr*-dependent fitness costs; thus, the fitness cost of *mcr* variants may differ in clinical strains adapted to *mcr*-carriage.

Our study also provides new insights on MCR-9, which has been of interest as it is frequently found in isolates classified as colistin susceptible (19), which may be explained by a recent observation that *mcr-9* expression in clinical isolates is generally low (36). Although several studies did not show increased MICs of strains naturally carrying or expressing *mcr-9* (18, 19), Kananizadeh et al. (17) found that *mcr-9*-positive *Enterobacter cloacae* isolates are resistant to colistin when grown in brain-heart infusion, tryptic soy, and lysogenic broth (MICs of 4 to 128 µg/mL), but not when grown in cation-adjusted Müller-Hinton broth (MHII; MICs of 0.125–0.5 µg/mL). Although we found similar MICs in cation-adjusted MHII, we found that MCR-9 expression conferred fitness advantages in the presence of subinhibitory levels of colistin in a competition assay. Although MCR-9 thus does not seem to confer resistance detectable in standard MIC assays, it does appear to provide fitness advantages in the presence of colistin, which suggests that *mcr-9* presence can be clinically relevant.

Importantly, our data showed that overexpression of the four canonical PETs resulted in significant fitness costs in the absence of colistin despite providing fitness advantages in the presence of colistin. This suggests that tight control of PET expression is essential, particularly for highly toxic *pet* genes like *eptA* and *mcr-9*. Indeed, *eptA* expression is tightly regulated by two-component systems PhoPQ and PmrAB in *Salmonella* (37). Although the regulation of *mcr-9* has not been well elucidated, a previous study reported an inverted repeat motif as a putative regulatory element found upstream of 95% of evaluated *mcr-9* genes (16); this motif may facilitate regulation. Interestingly, we also found that MCR-9 protein levels were significantly lower than MCR-1 and -3 levels when induced with the same L-arabinose concentration. This suggests that PETs are likely post-transcriptionally controlled through mRNA or protein stability, which has not yet been explored in the context of PETs. Exploring mRNA and protein stability of different PETs and their context-dependent fitness advantages in different environments (e.g., host environments, low ion conditions) may provide further insight into this group of enzymes.

We also saw that fitness in the presence of colistin was highly variable for strains expressing EptA or MCR-9. Protein levels in heterologous expression systems have been reported to vary across different cells in the same population (38), suggesting that expression of these two PETs likely generates subpopulations that differ in their relative fitness in this study. Our observations suggest that natural populations of *mcr*-carrying bacteria may engage in microbial bet-hedging (39, 40) to help manage the high fitness costs of some *mcr* variants. Although this strategy may apply to all *mcr*-carrying strains, our results suggest that this strategy is primarily utilized by strains expressing *pet* variants with higher cell viability defects and fitness costs (i.e., *eptA* and *mcr-9*), as *pet* variants with lower fitness costs demonstrated consistent fitness advantages in the presence of subinhibitory levels of colistin.

To expand our understanding of PETs, we also used our expression system to characterize two novel, *mcr-*like PETs. Both genes did not decrease colistin susceptibility or increase colistin tolerance in *E. coli* in MIC or killing assays, respectively, and appear to be phenotypically distinct from bona fide PETs by showing moderate cell viability defects. Although both PET-B and -C expressing strains showed fitness cost in the absence of colistin, neither showed fitness advantages in the presence of subinhibitory levels of colistin. Although the fitness cost conferred by PET-B in competition assays in the absence of colistin was not as high as fitness costs conferred by other PETs, the more bimodal distribution in fitness values and borderline statistically significant *P*-value suggest that some fitness costs were not detected in the competition assay.

As PET-B and -C have predominantly been identified in *P. aeruginosa* (5), these proteins may have substrate specificity for lipid A produced by *P. aeruginosa* and, hence, not confer resistance in *E. coli*. This idea is supported by reports of lipid A structural differences between organisms (28) and the fact that we were unable to detect pEtN-modification of lipid A by PET-B. The limited evidence for fitness defects in competition assays may also be linked to the lack of detectable lipid A modifications. However, we identified PET-C-mediated pEtN-modification of the 4′-phosphate of lipid A, which is the same residue that MCR-1 and -3 modify. The lack of detectable col^R^ in the PET-C expressing strain could be explained by the reduced function of PET-C in *E. coli,* such that the amount of modified lipid A was not sufficient to create measurable phenotypes. As a different study (41) showed that expression of a PET enzyme related to PET-C in *P. aeruginosa* conferred col^R^ and decreased cell viability, our results suggest caution when using heterologous hosts to detect col^R^ or changes in colistin susceptibility conferred by more diverse PET variants.

Additionally, some of the observed fitness defects may have been caused by codon bias of PET-B and -C. Although the codon adaption index (CAI) provides a good estimate of whether expression of heterologous proteins will be successful (42), that is, a higher CAI suggests ideal expression, with values ranging from 0 to 1 (43), studies have also shown that overexpressing genes with low CAIs (i.e., many rare codons are present) results in higher fitness costs to the expressing strains than overexpressing the same genes with high CAIs (44, 45). This is explained by the fact that the presence of rare codons reduces the speed of elongation, causing ribosomes to be sequestered by mRNAs for longer, thus reducing the overall pool of ribosomes and overall cellular resources, which results in decreased fitness (44, 45). Our western blot images indicate reliable expression of both PET-B and -C, which is validated through the CAI values of *petB* and *petC* genes when compared with the *E. coli* genome (i.e., 0.74 and 0.76, respectively) (46). By comparison, the CAI values of *mcr-1*, *-3*, and *-9* are 0.69, 0.63, and 0.7, respectively (46), suggesting that codon bias should not selectively impact *petB* and *petC* expression or fitness costs experienced by expressing cells.

We performed a comparative structural analysis to understand PET-B’s inability to modify *E. coli*’s lipid A. Although the correlation between the structural variability and the ability of PETs to modify lipid A should be cautiously viewed, it is tempting to speculate on the role of the structurally variable hinge regions in enzyme function. For example, it has been shown that the bridging helix and extended loop play a critical role in the function of EptA by acting as a hinge that offers extensive conformational flexibility between the membrane-bound and the catalytic domains (29, 47, 48). Additionally, a study showed that exchanging the linker region connecting the membrane-bound and the catalytic domains from a non-functioning MCR-3 homolog with a linker region of a functional MCR-3 homolog restored its ability to decrease colistin susceptibility (49). Thus, structural variability in the bridging helix region might relate to PET-B’s inability to modify *E. coli*’s lipid A.

As several papers have suggested a role for MCR in resistance to different AMPs (11–13, 15), we also tested the sensitivity of PET-expressing strains to a set of cell wall stressors and AMPs. Interestingly, we saw increased EDTA sensitivity in EptA and MCR-9 expressing strains, which may suggest that 1-phosphate pEtN modified lipid A may be more susceptible to EDTA-dependent ion depletion. Although our preliminary ZOI experiments did not identify any inhibition by LL-37 or lysozyme for any strains, cecropin A, an AMP produced by insects (23), showed inhibition of all tested strains. Some PET-expressing strains showed marginally smaller cecropin A ZOIs than the EV and PET-B expressing strain, suggesting possible protective capabilities against cationic AMPs other than colistin. Further AMP sensitivity tests using a wider range of AMP concentrations and different testing methods, e.g., competition assays (13, 50), may thus be valuable.

Importantly, we show for the first time that PETs stereoselectively modify lipid A in *E. coli*. Although MCR-1, MCR-3, and PET-C selectively modify the 4′-phosphate of lipid A, EptA and MCR-9 modify the 1-phosphate. For our canonical PETs, which all come from *Enterobacteriaceae* (16, 51, 52), the modified site seems to correlate with phenotypic outputs of col^R^ and fitness costs. Specifically, 4′-phosphate modification is associated with decreased susceptibility to colistin and low toxicity, whereas the 1-phosphate modification is associated with no change in colistin MIC and high toxicity. Although previous studies have shown that other PETs modify different LPS structures (i.e., EptB and EptC, which modify phosphate residues of the inner core), this is the first report of stereoselectivity of enzymes that fall into the EptA/MCR clade. These observations suggest a site specificity of colistin action (i.e., targeting of the 4′-phosphate), which could be exploited to develop improved membrane-targeting antibiotics that may use targets other than the 4′-phosphate. Further experiments examining the positional selectivity of additional PETs, including in other bacterial strains, will help our understanding of both the evolution of col^R^ and the potentially different roles PETs play in bacterial physiology, especially when examining how bacteria may co-evolve to adapt to PET-dependent fitness costs.

Overall, our results indicate that PETs are more phenotypically diverse than previously assumed. The six different PETs tested in this study fall into three different groups based on their phenotypes in *E. coli*: (i) PETs that considerably decrease colistin susceptibility but confer moderate fitness costs (i.e., MCR-1 and -3), (ii) PETs that provide fitness advantages to subinhibitory levels of colistin but high fitness costs in the absence of colistin (i.e., EptA and MCR-9), and (iii) PETs that may not provide col^R^ and may carry moderate fitness costs (i.e., PET-C). Most importantly, our results suggest that the stereoselectivity of the pEtN-attachment to lipid A matters for phenotypic outputs of col^R^ and fitness.

## MATERIALS AND METHODS

### Strains, vectors, and growth conditions

*E. coli* Top10 strains were grown at 37°C, 200 rpm in Difco LB Lennox Broth (LB; Becton, Dickinson and Company [BD]; Franklin Lakes, NJ; cat. #240230). For *E. coli* strains carrying pBAD24 or pUC19 vectors, 100 µg/mL of ampicillin (AMP) was added to maintain the vectors.

### Choosing putative, novel *mcr*-like variants

To further test our expression system, we selected two putative, novel *mcr*-like variants (NCBI nucleotide accession numbers: NZ_RWZM01000001.1 [locus tag: IPC1619_RS00685; referred to as *petB*] and NZ_KI519076.1 [locus tag: Q027_RS12935; referred to as *petC*]) for cloning and phenotypic characterization in our heterologous host systems. These two genes were selected based on a phylogenetic study of *mcr* and related genes reported by Gaballa et al. (5); this study predicted five putative, novel groups of *mcr* genes. As two of these groups, groups B and C, contained the largest number of sequences (6 and 13, respectively), we selected one sequence from each group for these experiments; the other three groups only had two or three predicted members (5). All putative, novel *mcr*-like genes from groups B and C come from *P. aeruginosa* isolates. We selected the putative, novel *mcr*-like gene referred to as *petB* from group B because it had the most conserved amino acid sequence, that is, 100% sequence identity with four of the other predicted members of this class. We selected the putative, novel *mcr*-like gene referred to as *petC* from group C as this gene is closest to a possible consensus sequence. In comparison to group B, group C genes and proteins showed greater sequence diversity, with a total of eleven amino acids differing between putative MCR-like proteins in group C. For 10 of these amino acid residues, PET-C has the amino acid that is found in most of the isolates. For one amino acid residue (residue 497) PET-C encodes a serine, which is found in five of the 13 isolates (the other eight isolates encode a glycine instead). The strain *petB* was found in was isolated from a cystic fibrosis patient, whereas the strain that *petC* was found in was isolated from a foot wound, suggesting these putative, novel *mcr* genes may have clinical relevance. Finally, the geographical isolation location for both isolates is in the United States, which may make it easier to obtain the isolate for future studies if needed.

### Cloning of canonical *mcr* variants, *eptA,* and putative, novel *mcr*-like variants

*mcr-3.1* and *mcr-9.1* with 3× FLAG tags were PCR amplified with pBAD24_mcr3_f, pBAD24_mcr9_f, and pBAD24_FLAG_r, respectively, from previously created vectors, pET17b-*mcr-3* and pET17b-*mcr-9* (21), with *mcr-3.1* coming from isolate *S. enterica* FSL R9-3269 and *mcr-9* coming from *S. enterica* FSL R9-3274. Primer information can be found in Table S2. *mcr-1.1* (NCBI nucleotide accession: NZ_KX772778.1 [locus tag: HTI99_RS00160]), *eptA* from susceptible isolate *S. enterica* FSL R9-5409 (WGS available under SRX3031601), *petB*, and *petC* with C-terminal 3× FLAG tag were synthesized in the pFASTBac1 vector with EcoRI and SalI cut sites by ThermoFisher Scientific’s GeneArt Service (ThermoFisher Scientific; Waltham, MA). *mcr-3.1*, *mcr-9.1*, vector pBAD24 (American Type Culture Collection [ATCC]; Manassas, VA, cat. #87399), and pFASTBac1 vectors with *mcr-1*, *eptA, petB*, and *petC* genes were digested with restriction enzymes EcoRI and SalI (New England Biolabs [NEB]; Ipswich, MA), purified and ligated with T4 DNA ligase (NEB, cat. #M0202L) according to the manufacturer’s recommendations. The ligated vectors were transformed into *E. coli* Top10 (ThermoFisher, cat. #C404010) via heat shock, according to the manufacturer’s recommendations. Transformants were selected by plating on LB + 100 µg/mL Amp and overnight incubation at 37°C. Constructs were confirmed via Sanger sequencing and subsequent analysis in Geneious Prime (Auckland, New Zealand) using the universal pBAD24 forward and reverse sequencing primers (Table S2).

### Cell viability assessment

Cell viability of *pet* expressing strains was assessed by growing *E. coli* strains at 37°C and 200 rpm shaking in LB + 100 μg/mL AMP for 12–18 h, followed by 1:200 back dilution in BBL Mueller-Hinton II cation-adjusted broth (MHII, BD cat. #212322) + 100 µg/mL AMP and incubation until an OD_600_ of 0.25–0.4 was reached. Canonical *mcr* variants and *eptA* were induced with final L-arabinose (Sigma-Aldrich; St. Louis, MO, cat #A3256-100G) concentrations of 0%, 0.01%, 0.02%, 0.05%, 0.1%, or 0.2% (wt/vol) and grown at 37°C and 200 rpm shaking; putative, novel *mcr*-like variants were induced with final L-arabinose concentrations of 0% and 0.05% (wt/vol). Before induction and after 1, 4, 12, and 22 h of induction, 100 µL of each culture was removed. Ten-fold serial dilutions were prepared in phosphate-buffered saline (PBS), and 10 µL volumes were spotted onto LB Broth (BD, cat. #240230) agar plates (prepared with 15 g/L Bacto Agar [BD, cat. #214010]) and incubated at 37°C overnight. Colony forming units (CFU/mL) were calculated from colony counts, and log_10_-transformed data were plotted. Experiments were performed in biological triplicates. Log_10_-transformed data were analyzed by a two-way ANOVA and the emmeans package.

### Susceptibility testing

Susceptibility to colistin and EDTA was tested by broth microdilution (BMD) based on CLSI guidance (53) with the exception being the use of PlateOne polypropylene 96-well plates (USA Scientific; Ocala, FL; cat. #1837-9610) for colistin MIC assays because colistin has a higher binding affinity to polystyrene (54, 55). For EDTA assays, 96-well clear flat-bottom polystyrene microplates (Corning; Corning, NY; cat. #3370) were used. Briefly, strains were grown in LB + 100 µg/mL AMP at 37°C and 200 rpm shaking for 12–18 h and subsequently diluted 1:200 in MHII broth + 100 µg/mL AMP, followed by incubation at 37°C and 200 rpm shaking until cultures reached an OD_600_ of 0.25–0.4. Strains were induced by the addition of different concentrations of L-arabinose ranging from 0.01% to 0.2% (wt/vol), or filter-sterilized dH_2_O as a negative control and grown at 37°C and 200 rpm shaking for 1 h after arabinose addition. Strains were subsequently inoculated, at a final concentration of 5 × 10^5^ CFU/mL, into MHII broth containing colistin (0.03–32 µg/mL, 2-fold dilutions) (Sigma-Aldrich, cat #C4461-1G) and L-arabinose (0%, 0.01%, 0.02%, 0.05%, 0.1%, 0.2%) and grown statically for 16–20 h at 35°C. Each canonical *mcr* variant, as well as *eptA*, was tested at every L-arabinose concentration in biological triplicates. Putative, novel *mcr*-like variants were tested at 0% and 0.05% L-arabinose in biological triplicates. EDTA assays were performed with all PET-expressing strains at 0.05% L-arabinose (wt/vol) and EDTA (Fisher Bioreagents; Pittsburgh, PA, cat #BP120-1) concentrations ranging from 15.625 µg/mL to 16 mg/mL in 2-fold dilutions in MHII broth.

### Western blot analysis of heterologous protein expression

Western blot analysis was performed as previously described to test whether our heterologous PET-FLAG proteins were expressed (21). Briefly, *E. coli* strains were grown at 37°C, 200 rpm shaking in LB + 100 μg/mL AMP for 12–18 h, followed by 1:200 backdilution in MHII broth + 100 μg/mL AMP and incubation until an OD_600_ of 0.25–0.4 was reached. Cells were induced with 0.05%, 0.2% (wt/vol) L-arabinose, or dH_2_O as a negative control for 12 h and collected by centrifugation. Cells were lysed in SDS-PAGE lysis buffer by heating (95°C for 10 min) and sonication as detailed by Schumann et al. (21). Lysed cells were resolved on a 4% to 20% Mini-Protean TGX precast protein SDS-PAGE gel (Bio-Rad Laboratories; Hercules, CA, cat. #4568096), followed by protein visualization with the Bio-Rad ChemiDoc MP Imaging system after exposure UV light for 1.5 min. The proteins were transferred to polyvinylidene difluoride (PVDF) membrane and blocked with TTBS (TBS buffer [50 mM Tris-Cl, 150 mM NaCl, pH 7.5] with 0.1% [vol/vol] Tween 20) containing 5% Blotting-Grade Blocker (Bio-Rad Laboratories, cat. #170-6404) for 30 min. PVDF membranes were incubated with rabbit anti-FLAG primary antibody (Sigma-Aldrich, cat. #F7425, at 1:500 dilution) diluted in TTBS with 0.5% (wt/vol) skimmed milk powder, at room temperature (RT) overnight with gentle shaking, followed by three 10 min washes with TTBS. PVDF membranes were incubated with goat anti-rabbit-horseradish peroxidase secondary antibody and subsequently developed and visualized as previously detailed (21). To analyze levels of PET-FLAG proteins semi-quantitively, the area under the curve was estimated for PET-FLAG bands and all protein bands on the SDS page gels via densitometric analysis of band intensity using the Bio-Rad Image Lab 6.1 software as previously described (21). Data were analyzed using a one-way ANOVA with a post-hoc Tukey test.

### Construction of maximum likelihood phylogeny of *mcr* and *mcr*-like genes

Maximum likelihood (ML) phylogeny was inferred from the nucleotide sequences of *E. coli* K-12 substr. MG1655 *eptA*, *eptB,* and *eptC* genes, representatives of *mcr* families, and the newly identified *petB* and *petC* genes. The coding sequences of *eptA*, *eptB,* and *eptC* were extracted from WGS of the *E. coli* K-12 substr. MG1655 (accession number NC_000913.3). Representative of *mcr*-1 to *mcr*-10 families were selected from a previously reported *mcr* phylogeny (5); briefly, phylogenetic outlier variants in each *mcr* family were excluded (e.g., *mcr*-2.4 and *mcr*-3.17), and the first reported variant in each family was selected. The coding sequences of *mcr*-1.1 (accession number NG_050417.1), *mcr*-2.1 (accession number NG_051171.1), *mcr*-3.1 (accession number NG_055505.1), *mcr*-4.1 (accession number NG_057470.1), *mcr*-5.1 (accession number NG_055658.1), *mcr*-6.1 (accession number NG_055781.1), *mcr*-7.1 (accession number NG_056413.1), *mcr*-8.1 (accession number NG_061399.1), *mcr*-9.1 (accession number MK070339.1), and *mcr*-10.1 (accession number NG_066767.1), *E. coli eptA*, *eptB,* and *eptC*, and the newly identified *petB* and *petC* genes were used to construct back-translated nucleotide multiple sequence alignments (NT_btn_-MSA) using MUSCLE (56) with the default settings in Geneious version 2019.2.3 (Biomatters, Auckland, New Zealand). The resulting NT_btn_-MSAs were used to construct ML phylogenies with 100 bootstrap replicates via RAxML, using the GTRGAMMA substitution model and default settings (RAxML GUI version 2.0.10 and RAxML version 8.2.12) (57). The resulting trees were visualized and edited using iTOL version 6.5 (58).

### Sample collection for mass spectrometry

*E. coli* strains with the pBAD24 vectors were grown at 37°C and 200 rpm shaking in LB + 100 µg/mL AMP for 12–18 h, followed by 1:200 backdilution in MHII broth + 100 µg/mL AMP and incubation until an OD_600_ of 0.25–0.4 was reached. Canonical *mcr* variants and *eptA* were induced with final L-arabinose concentrations of 0%, 0.05%, or 0.2% (wt/vol) and grown at 37°C and 200 rpm shaking; putative, novel *mcr*-like variants were induced with the final L-arabinose concentrations of 0% or 0.05% (wt/vol). Cells were collected from 1.5 mL aliquots before induction and 1, 4, 12, and 22 h post-induction by centrifugation at 8,000 rpm for 3 min (Eppendorf 5417C centrifuge) and from 3 mL aliquots 4 h post-induction by centrifugation at 7,197 × *g* for 5 min (Sorvall X4RF PRO-MD centrifuge). Cells were collected from 1.5 mL (0, 1, 2 h) and 3 mL aliquots (4 and 12 h) by centrifugation at 8,000 rpm for 3 mins (Eppendorf 5417C centrifuge). The supernatant was decanted, and the pellets were stored at −80°C and shipped to Dr. Robert Ernst on dry-ice.

### Mass spectrometry

A Bruker microFlex and MALDI (tims TOF) MS were used for FLAT and FLAT^n^ experiments (26, 59). The MALDI (tims TOF) MS was equipped with a dual ESI/MALDI source with a SmartBeam 3D 10 KHz frequency tripled Nd:YAG laser (355 nm). The system was operated in “qTOF” mode (TIMS deactivated). Ion transfer tuning was used with the following parameters: Funnel 1 RF: 440.0 Vpp, Funnel 2 RF: 490.0 Vpp, Multipole RF 490.0 Vpp, is CID Energy: 0.0 eV, and Deflection Delta: −60.0 V. Quadrupole was used with the following values for MS mode: Ion Energy: 4.0 eV and Low Mass 700.00 *m*/*z*. Collision cell activation of ions used the following S-4 values for MS mode: Collision Energy 9.0 eV and Collision RF 3900.0 Vpp. In the MS/MS mode, the precursor ion at *m*/*z* 1919.22 was chosen. Isolation width and collision energy were set to 4 *m*/*z* and 110 eV, respectively. Focus Pre TOF used the following values for Transfer Time: 110.0 µs and Pre Pulse Storage 9.0 µs. Agilent ESI Tune Mix was used to perform calibration of the *m*/*z* scale. MALDI parameters in qTOF were optimized to maximize intensity by tuning ion optics, laser intensity, and laser focus. All mass spectra were collected at 104 µm laser diameter with beam scan on using 800 laser shots per spot and 80% laser power, respectively. Both MS and MS/MS data were collected in negative ion mode. A microFlex was used as a comparison to MALDI (tims TOF) MS. In all cases, 10 mg/mL of norharman (NRM)1,4 in 1:2 MeOH:CHCl_3_ (vol:vol) was used for lipid A detection. NRM solution (1 µL) was deposited on the sample spot.

For data processing, all MALDI (timsTOF) MS and MS/MS data were visualized using mMass (Ver 5.5.0). Peak picking was conducted in mMass. Identification of all fragment ions was performed with Chemdraw Ultra (Ver10.0).

### Killing assays

*E. coli* Top10 strains with the pBAD24 vectors were grown at 37°C and 200 rpm shaking in LB + 100 µg/mL AMP for 12–18 h, followed by 1:200 backdilution in MHII broth + 100 µg/mL AMP and incubation until an OD_600_ of 0.25–0.4 was reached. All *pet-*expressing strains were induced with a final concentration of 0.05% (wt/vol) of L-arabinose for 1 h at 37°C and 200 rpm shaking. Culture OD_600_s were adjusted to 0.6–0.8, followed by splitting each culture into five aliquots and colistin addition at final concentrations of 0, 1, 2, 4, or 8 µg/mL; these cultures were further grown statically at 35°C for 1 h. Afterward, 10-fold serial dilutions were prepared in PBS, and 10 µL volumes were spot-plated onto LB agar plates and incubated at 37°C overnight. Colony forming units (CFU/mL) were calculated from colony counts, and the relative log reduction in comparison to the control (grown in 0 µg/mL of colistin) was calculated for each strain and colistin concentration. Experiments were performed in biological triplicates.

### Competition assays

*E. coli* Top10 (“WT”) was used as a control to calculate the relative fitness of each PET-expressing strain. *E. coli* top 10 strains competed against *E. coli* top 10 pUC19, which produces blue pigment when grown on media containing 5-bromo-4chloro-3-indoyl β-D-galactopyranoside (X-Gal) (Gold Biotechnology; Olivette, MS, cat # X4281C10). *E. coli* top 10 strains were grown at 37°C and 200 rpm shaking for 12–18 h, followed by 1:200 back dilution in MHII broth + 100 µg/mL AMP for pBAD24 and pUC19 carrying strains until an OD_600_ of 0.25–0.4 was reached. All strains were induced with a final concentration of 0.05% (wt/vol) of L-arabinose for 1 h at 37°C and 200 rpm shaking. *E. coli* Top10 or *E. coli* Top10 pBAD24 strains were added to fresh MHII media containing 0.05% L-arabinose at a 1:1 ratio with competitor strain *E. coli* Top10 pUC19 at a final concentration of 2.5 × 10^5^ CFU/mL. We plated 10-fold serial dilutions of all strains before the competition to determine initial concentrations. Competition experiments were carried out for 12 h at 37°C and 200 rpm, followed by plating 10-fold serial dilutions made in PBS on LB plates supplemented with 20 µg/mL of X-Gal. Plates were incubated overnight at 37°C, and white colonies and blue colonies were counted in the countable range (20–200 colonies/plate). We used the following formula to calculate fitness:

$$fitness=\frac{final\;concentration\;(white)/initial\;concentration\;(white)}{final\;concentration\;(blue)/initial\;concentration\;(blue)}$$

We chose to not log transform the white or blue cell ratios when calculating fitness values to avoid reporting negative fitness values (due to die-off of some strains), which could be misinterpreted. For competitions in the presence of colistin, cells were washed in PBS before the competition assay and colistin was added to the MHII media at concentrations of one-fourth of the PET expressing strain’s MIC (i.e., 0.0625 µg/mL for WT, pBAD24, pBAD24-*eptA*, -*mcr-9*, *-petB*, and *-petC*, 0.5 µg/mL for pBAD24-*mcr-3*, and 1 µg/mL for pBAD24-*mcr-1*). Competition assays were performed in biological quadruplicates and quintuplicates in the absence and presence of colistin, respectively. For the competition assays in the presence of colistin, we added one colony to each count because we could not recover cells for some of the assays. Statistics were performed on log_10_-transformed fitness values; means were compared by a one-way ANOVA with post-hoc Dunnett’s test or Kruskal-Wallis test with post-hoc Many-to-One Dunn’s test. Variances of fitness values for competition assays performed in the presence of colistin were compared by calculating the absolute differences between log_10_-transformed fitness values and their group’s median, followed by a one-way ANOVA with a post-hoc Tukey test.

### Zone of inhibition assays

For the zone of inhibition assays, MHII plates containing 0.05% L-arabinose were prepared. *E. coli* Top10 pBAD24 strains were grown overnight in LB + 100 µg/mL AMP at 37°C, 200 rpm shaking, diluted 1:20 in 3 mL of MHII Top agar (0.75% agarose) containing 0.05% L-arabinose and spread on MHII plates. Disks made from autoclaved filter paper (Whatman; Maidstone, UK, cat #1001090) were added to plates with sterile forceps and the following compounds were added in 10 µL volumes with final amounts (per disk) listed in parenthesis: colistin (10 µg) (Sigma-Aldrich, cat C4461-1G), nisin (10 µg) (Sigma-Aldrich, cat #N5764-5G), LL-37 (10 µg) (Sigma-Aldrich, cat #94261-1MG), cecropin A (10 µg) (Sigma-Aldrich, cat #C6830-.5MG), lysozyme (10 µg) (Sigma-Aldrich, cat #62971-10G-F), sodium dodecyl sulfate (1%) (J. T. Baker, Phillipsburg, NJ, cat #4095-02), distilled water, and distilled water (pH 2). Plates were incubated for 24 h at 35°C and zones of inhibition were measured and recorded. Experiments were performed as biological duplicates.

### Protein structural modeling

Structural modeling of PET proteins was done using AlphaFold2 (60) as implemented in the ColabFold v1.5.5 (61) using the default setting and the structure of the *Neisseria meningitidis* phosphoethanolamine transferase EptA structure as a template (48). The structures were viewed and annotated using UCSF ChimeraX V 1.6.1 (62). Structural relationships between different PET proteins were assessed using the DALI server (63). Structural similarity matrices and cluster dendrograms are based on the DALI Z-score comparisons calculated from a DALI all-against-all analysis (64). Quality assessment and superimposition of protein structures were done using the Swiss-Model Qualitative Model Energy Analysis (QMEAN) server and Structure Comparison tool (65). Superimposed structures were downloaded, viewed, and edited in UCSF ChimeraX V 1.6.1(62).

### Statistical analysis

Data were analyzed using R Statistical Software (Version 4.1.2; R Foundation for Statistical Computing, Vienna, Austria).

## Data Availability

All raw data files are available on GitHub (https://github.com/as-2635/PETs_colR_tox) or can be made available on request.

## SUPPLEMENTAL MATERIAL

Supplemental material msphere.00731-24-s0001.docx Fig. S1 to S5; Tables S1 and S2.

ASM does not own the copyrights to Supplemental Material that may be linked to, or accessed through, an article. The authors have granted ASM a non-exclusive, world-wide license to publish the Supplemental Material files. Please contact the corresponding author directly for reuse.
