# Supplementary material for: Site-selective modifications by lipid A phosphoethanolamine transferases linked to colistin resistance and bacterial fitness
Source: mSphere. 2024 Nov 29;9(12):e00731-24. doi: 10.1128/msphere.00731-24 (PMC11656738; doi:10.1128/msphere.00731-24)
Supplement: Supplemental material — Fig. S1 to S5; Tables S1 and S2. [file msphere.00731-24-s0001.docx]

**Figure S1.** **SDS Page gels and western blots of PET variants.**

Each column represents the SDS Page gel used to determine total protein and its corresponding western blot used to determine the protein level for heterologously expressed PET variants. Each gel and blot represent one set of biological replicates (n=3).

**Figure S2.** **Cell viability graphs after induction with different L-arabinose concentrations.**

Graphs show cell viability counts across 22 hours post-induction with different L-arabinose concentrations. The data collected after 4 and 12 hours of induction were used for Fig. 1B, and C. Data represent means, n = 3 ± SEM

**Figure S3.** **Protein expression of putative, novel MCR variants.**

SDS Page gel (left) and western blot (right) used to determine whether PET-B and PET-C were expressed. Each lane corresponds to one set of a biological replicate (n=3).

| **PET expressed** | **pEtN modification of lipid A after induction with L-arabinose^a^** | | | | | | | | | | | | | | | |
| --- | --- | --- | --- | --- | --- | --- | --- | --- | --- | --- | --- | --- | --- | --- | --- | --- |
|  | 0 hrs | |  | 1 hrs | | 4 hrs | | | |  | | 12 hrs | |  | 22 hrs | |
|  | 0% | 0.05% |  | 0% | 0.05% | |  | 0% | 0.05% | |  | 0% | 0.05% |  | 0 % | 0.05 % |
| EV |  |  |  |  |  | |  |  |  | |  |  |  |  |  |  |
| EptA |  |  |  |  | x | |  |  | x | |  | x | x |  |  |  |
| MCR-1 | x | x |  | x | x | |  | x | x | |  | x | x |  |  |  |
| MCR-3 | x |  |  | x | x | |  | x | x | |  | x | x |  | x | x |
| MCR-9 |  |  |  | x | x | |  | x | x | |  | x | x |  |  |  |
| PET-B |  |  |  |  |  | |  |  |  | |  |  |  |  |  |  |
| PET-C |  |  |  |  | x | |  |  | x | |  |  | x |  |  |  |

**Table S1.** **PEtN modification of lipid A throughout time and different L-arabinose concentrations**

^a^An "x" indicates the detection of pEtN modifications at a given time point and L-arabinose concentration (n=1).

**Figure S4. Relative quantitation of modification site by pEtN.**

(**A**) A bar graph of the ratio of intensities of two diagnostic ions between 1267.8095 and 833.4335 represents 4’- and 1-phosphate group modification by PEtN addition, respectively. The data represent means, n = 2 ± SD. (**B**) A list of the percentage of modification sites between the 1 and 4’-phosphate groups. MCR-1, MCR-3, and PET-C highly prefer 4’-phosphate group modification. The other two strains, MCR-9 and EptA, selectively modify the 1-phosphate group with 75%.

**Figure S5.** **PETs share an overall domain structure but diverse localized structures.**

PET-B, PET-C, MCR-1 to MCR-10, EptA, EptB and EptC structural models were predicted using AlphaFold2 based on the *Neisseria meningitidis* phosphoethanolamine transferase EptA structure as a template (NCBI structure accession number 5FGN). The Dali server was used to perform all-against-all comparisons of 3D protein structural models. (**A**) Structural similarity dendrogram, (**B**) similarity matrix, and (**C**) correspondence analysis plots were constructed based on the DALI Z-score.

**Table S2.** **Primers used in this study**.

| **Primer name** | **Sequence** |
| --- | --- |
| AS2635_1_pBAD24_mcr3_f | 5' GCATGAATTCACCATGAGTGATGTCTC 3' |
| AS2635_2_pBAD24_mcr9_f | 5' GCATGAATTCACCATGTTTTTACTGGTTTAC 3' |
| AS2635_3_pBAD24_FLAG_r | 5’ CCAT GTCGACTTATTTATCATCATCATC 3´ |
| AS2635_4_pBAD24_seq_f | 5' ATGCCATAGCATTTTTATCC  3' |
| AS2635_5_pBAD24_seq_r | 5' GATTTAATCTGTATCAGG 3' |
